# Multiple surgical interventions improve survival in locally recurrent retroperitoneal sarcoma: a retrospective cohort analysis

**DOI:** 10.3389/fonc.2025.1575260

**Published:** 2025-07-31

**Authors:** Guojun Yan, Xinbao Li, Kai Zhang, Chao Gao, Lijun Yan, Xinjing Zhang, Songlin An, Yanbin Zhang

**Affiliations:** Department of Peritoneal Cancer Surgery, Beijing Shijitan Hospital, Capital Medical University, Beijing, China

**Keywords:** retroperitoneal sarcoma, surgical interventions, survival outcomes, oncological prognosis, recurrent disease

## Abstract

**Background:**

Locally recurrent retroperitoneal sarcoma (RPS) poses substantial clinical challenges, especially in patients experiencing multiple recurrences. This study aims to evaluate the survival benefits conferred by repeated surgical resections in the management of recurrent RPS.

**Methods:**

A retrospective cohort study was conducted involving 56 patients who underwent repeated surgical resections for locally recurrent RPS at our institution between June 2016 and September 2023. Demographic, clinical, and histopathological variables- including age, sex, tumor differentiation, FNCLCC grade, prior radiotherapy and chemotherapy, surgical margin status, and postoperative complications -were collected and analyzed. Survival outcomes were assessed using Kaplan-Meier estimates and Cox proportional hazards models.

**Results:**

The median age of the cohort was 53 years (range: 28–72), with a male-to-female ratio of 30:26. Compared to a single resection, median overall survival (OS) improved with successive surgeries: 79.3 months for two surgeries, 158.0 months for three, and 181.7 months for four. However, OS declined to 121.9 months following five resections. Tumor differentiation and FNCLCC grade were significantly associated with survival outcomes. Multivariate analysis identified age, pathological subtype, tumor grade, and number of surgeries as independent prognostic factors. Although severe adverse events (SAEs) were recorded, no 30-day postoperative mortality occurred.

**Conclusions:**

Repeated surgical resection appears to confer substantial survival benefits in patients with locally recurrent RPS, underscoring the clinical value of surgical management in selected cases. These findings highlight the importance of individualized surgical strategies, while emphasizing the need for further investigation to optimize treatment paradigms.

## Introduction

The mean annual incidence of RPS is approximately 0.5 to 1 case per 10,000 individuals. The management of RPS constitutes a significant challenge in oncological practice, necessitating a nuanced understanding of both its biological behavior and the complex anatomy of the retroperitoneal space (1–5). RPS, a rare malignancy arising in the retroperitoneum -the anatomical space located posterior to the retroperitoneum lining that encases various vital organs -requires meticulous and strategic intervention due to its proximity to critical structures. Surgical resection has long been regarded as the cornerstone of effective treatment for RPS, offering the most chance for long-term disease control, particularly in cases of localized disease where complete resection can substantially improve survival outcomes (4, 6–9).

Despite the potential curative benefit of initial surgical resection, disease recurrence remains a common and formidable obstacle. Reported recurrence rates range from 20% to 55%, with considerable variability depending on histological subtype and anatomical site of recurrence. In particular, primary dedifferentiated liposarcomas (DDLPS) exhibit local recurrence rates as high as 80%, highlighting the aggressive clinical behavior of this subtype and the difficulty in achieving durable disease control (10–12).

Recurrent RPS presents distinct therapeutic challenges. Reoperations are associated with increased technical complexity and higher risks of perioperative morbidity, necessitating a careful assessment of the risk-benefit profile of repeated interventions. Moreover, prior surgeries can significantly alter anatomical planes, complicating subsequent resections and potentially compromising outcomes. The oncological benefit of repeated surgical resection in locally recurrent RPS remains a subject of debate. While some studies suggest improved survival and local control with multiple resections, others emphasize the elevated perioperative risks and potential detriment to the patient’s quality of life. The therapeutic efficacy of such interventions is likely influenced by factors including histological subtype, extent of prior treatments, and the patient ‘s general health status.

Given the rarity of RPS and the inherent complexity of managing locally recurrent disease, high-quality evidence to guide clinical decision-making remains scarce. This study aims to evaluate the efficacy of repeated surgical interventions in patients with locally recurrent RPS, with particular attention to oncological outcomes, perioperative morbidity, and overall survival. By offering a comprehensive analysis of the benefits and limitations of iterative surgical management in this context, we seek to inform clinical practice and support the development of evidence-based therapeutic strategies.

## Methods

This retrospective cohort study analyzed clinical data from patients with locally recurrent RPS who underwent iterative surgical interventions at Beijing Shijitan Hospital between June 2016 and September 2023. The study was conducted in full accordance with the ethical standards of the institutional research committee and conformed to the principles outlined in the 1964 Declaration of Helsinki and its subsequent amendments. Ethical approval was obtained from the Ethics Committee of Beijing Shijitan Hospital. Written informed consent was acquired from all participants undergoing repeated surgical procedures, following comprehensive disclosure of the surgical approach and associated risks.

Inclusion criteria were as follows: (1) histopathologically confirmed RPS with a prior history of surgical resection; (2) radiologically confirmed local recurrence based on computed tomography (CT) or positron emission tomography (PET) imaging; (3) multidisciplinary consensus - comprising surgical, medical oncology, radiology, and pathology experts-deeming reoperation both feasible and beneficial; (4) resectable lesions as determined by preoperative imaging; and (5) adequate organ function and performance status (Eastern Cooperative Oncology Group [ECOG] score ≤2) sufficient to tolerate major surgery.

Exclusion criteria included: (1) patients undergoing initial resection for primary RPS without prior surgical history; and (2) unresectable disease or medical contraindications to surgery such as severe cardiopulmonary impairment. On the other hand, unresectability was determined by multidisciplinary tumor board (MDT) consensus based on: Radiological criteria: Involvement of >2 major vascular structures (e.g., SMA/SMV roots, aorta), or diffuse peritoneal seeding; ECOG PS ≥3, or severe cardiopulmonary comorbidities contraindicating major surgery; Cases deemed borderline resectable on imaging but found unresectable during diagnostic laparoscopy were classified as “unresectable.”

The following clinical and pathological variables were collected and analyzed: age, sex, histological subtype, Fédération Nationale des Centers de Lutte Contre le Cancer (FNCLCC) tumor grade, resection margin status (R0/R1 vs R2), use of radiotherapy and chemotherapy, presence of pathological organ invasion, and performance of contiguous organ resection. Notably, FNCLCC grading and other pathological features were recorded based on the findings from the most recent surgical specimen in each patient’s treatment course. The extent of resection was categorized as complete gross resection (R0/R1) or incomplete resection (R2). Tumor grading was performed according to the FNCLCC system, which incorporates tumor differentiation, extent of necrosis, and mitotic activity. Tumor recurrence was defined as radiologically confirmed by reappearance of disease following resection. Reoperation was performed when recurrence was deemed surgically manageable. Patients were stratified according to the number of Surgical interventions received. OS was evaluated in relation to both the frequency and timing of surgeries.

Postoperative complications were classified using the Clavien-Dindo system, which categorizes 48 distinct adverse events into nine grades. SAEs were defined as those corresponding to Grades III and IV (13).

### Statistical analysis

Continuous variables summarized as medians with interquartile ranges (IQRs), while categorical variables were expressed as counts and percentages. All statistical analyses were performed using SPSS software, version 25.0 for Windows (IBM Corp., Armonk, NY, USA). A two-sided *P* value of < 0.05 was considered statistically significant. OS was estimated using the Kaplan-Meier method. Univariate analyses of prognostic factors influencing OS were conducted using the log-rank test, and variables with *P* < 0.10 were subsequently included in a multivariate analysis using the Cox proportional hazards regression model To identify independent prognostic factors. Patients were stratified into groups based on the number of surgical resections (2, 3, 4, or ≥5) to reflect clinically relevant decision-making thresholds and to ensure sufficient sample sizes for meaningful comparative analysis.

## Results

A total of 56 patients with locally recurrent RPS underwent multiple surgical interventions between June 2016 and September 2023. The median age at the time of the final surgery was 53 years (range: 28–72 years), with a male-to-female distribution of 30:26 (53.6% vs. 46.4%).

Histologically, dedifferentiated liposarcoma (DDLPS) was the predominant subtype, accounting for 62.5% of cases (n=35), followed by well-differentiated liposarcoma (WDLPS), observed in 37.5% (n=21). According to the FNCLCC grading system, tumors were classified as grade I in 33.9% (n=19), grade II in 30.4% (n=17), and grade III in 30.4% (n=17), while grading data were unavailable for 5.4% of patients (n=3).

With regard to surgical frequency, the majority of patients underwent two resections (55.4%, n=31), followed by three (23.2%, n=13), four (8.9%, n=5), and between five and eight surgeries (12.5%, n=7). Detailed demographic, clinicopathological, and treatment characteristics are summarized in **Table 1**.

**Table 1:** Demographic, clinicopathological and treatment characteristics of patients.

At the time of last operation			
Variables	Median [Range]	n	%
Age, median	53 [28 ~72]		
< 60		39	69.60%
≥ 60		17	30.40%
Sex			
Female		26	46.40%
Male		30	53.60%
Histological subtype			
WDLS		21	37.50%
DDLS		35	62.50%
FNCLCC grade			
I		19	33.90%
II		17	30.40%
III		17	30.40%
Not available		3	5.40%
Frequency of surgery			
2		31	55.40%
3		13	23.20%
4		5	8.90%
5~8		7	12.50%
Combined organ resection (first surgery)			
None		35	62.50%
Single organ		11	19.60%
Multiple organs		10	17.90%
Organ resection (last surgery)			
Colectomy		32	57.10%
Splenectomy		6	10.70%
Gastrectomy		2	3.60%
Small bowel resection		20	35.70%
Hepatectomy		1	1.90%
Nephrectomy		9	16.10%
Pancreatectomy		7	12.50%
Diaphragm resection		6	10.70%
Cystectomy		1	1.90%
Radiotherapy throughout the disease trajectory			
Yes		8	14.30%
No		47	83.90%
Chemotherapy throughout the disease trajectory			
Yes		28	50.00%
No		28	50.00%
Margin status			
R0/1		45	80.40%
R2		11	19.60%

WDLPS, well-differentiated liposarcoma; DDLPS, dedifferentiated liposarcoma; FNCLCC, Fédération Nationale des Centers de Lutte Contre le Cancer.

During the initial surgery, 35 patients underwent gastrectomy without combined organ resection, 11 patients underwent resection of a single additional organ, and 10 patients underwent resection of multiple additional organs. The resected organs included the gallbladder, uterus, fallopian tubes and ovaries, colon, small intestine, spleen, diaphragm, kidney, adrenal gland, and vascular reconstruction.

### Overall survival

Taking the first surgery as the reference point, OS improved with successive surgical interventions: 79.3 months following two surgeries, 158.0 months after three surgeries, and 181.7 months after four surgeries. However, OS declined to 121.9 months in patients who underwent five or more surgeries.

**Figure 1:**
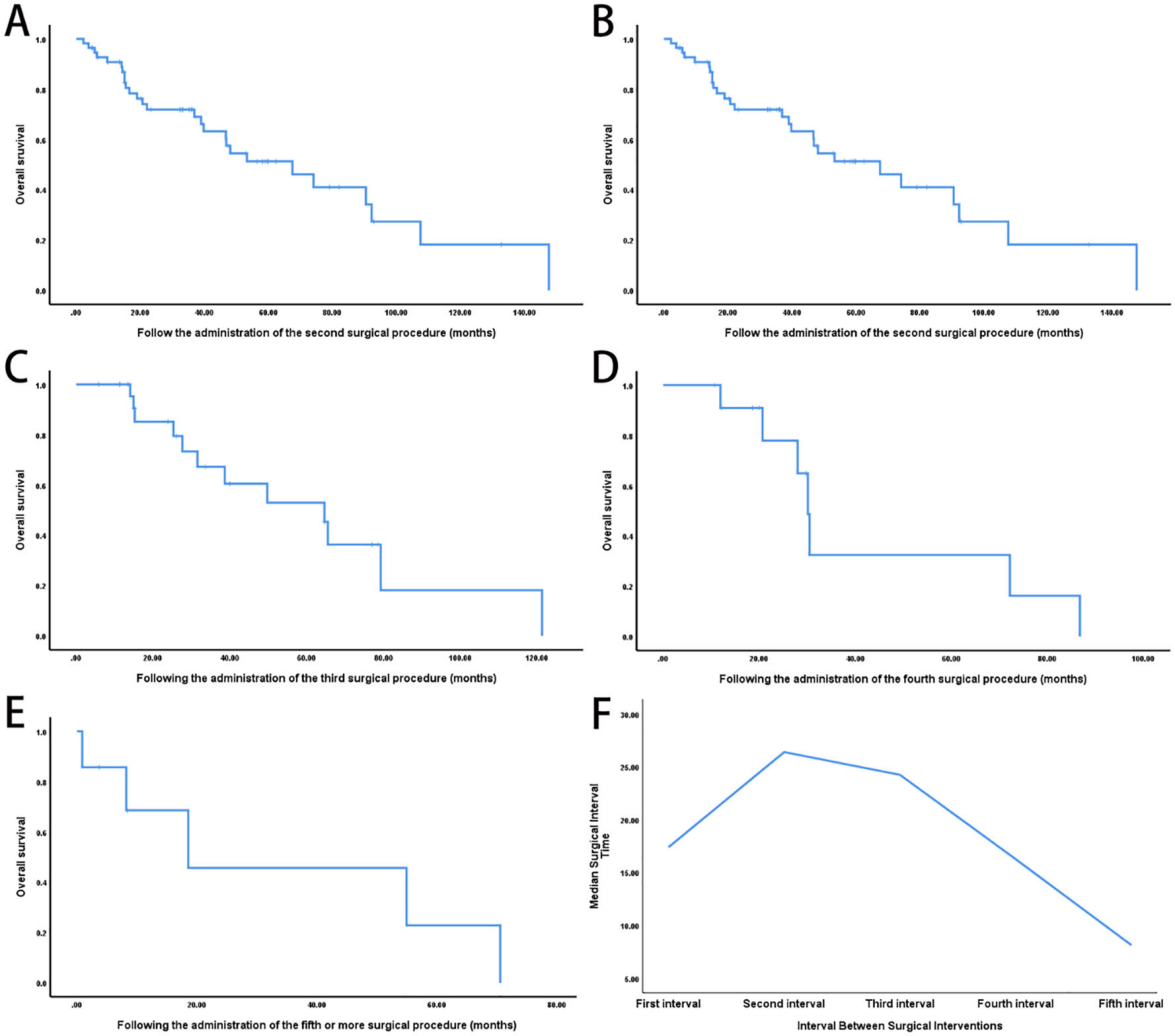
Impact of repeated surgical interventions on survival outcomes in recurrent retroperitoneal sarcoma. **(A)** Kaplan-Meier OS curve from the time of the first surgery (n = 56; median OS: 104.3 months). **(B)** OS following the second surgery for local recurrence (n = 31, median OS: 67.5 months). **(C)** OS after the third surgery (n = 13; median OS: 64.6 months). **(D)** OS after the fourth surgery (n = 5; median OS: 30.1 months). **(E)** OS in patients who underwent five or more surgeries (n = 7; median OS: 18.5 months). **(F)** Progressive shortening of recurrence-free intervals with increasing number of surgeries (log-rank P < 0.001).

Survival duration following each recurrence showed a decreasing trend: the median OS was 104.3 months after the first surgery, 67.5 months after the second, 64.6 months after the third, 30.1 months after the fourth, and 18.5 months after five or more surgeries (**Figures 1A–E**). In parallel, the interval between surgeries progressively shortened with each successive recurrence (**Figure 1F**).

**Figure 2:**
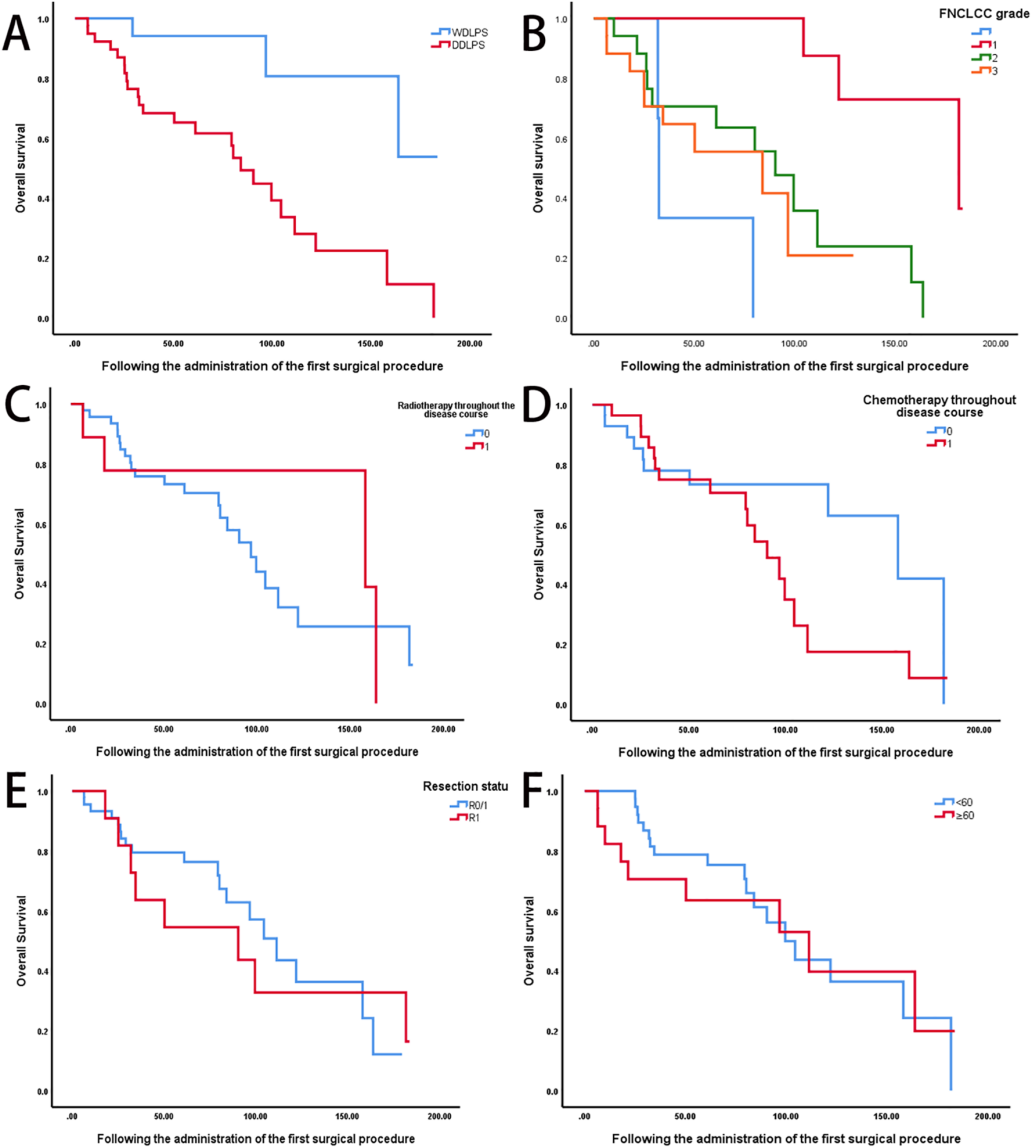
Prognostic factors influencing survival outcomes in recurrent retroperitoneal sarcoma. **(A)** Comparison of OS between well-differentiated liposarcoma (WDLPS, n = 21) and DDLPS, n = 35) (log-rank P < 0.05). **(B)** OS stratified by FNCLCC grade (Grade I: n=19, median OS: 181.7 months; Grade II: n=17, 90.3 months; Grade III: n=17, 84.0 months; P < 0.05). **(C)** OS according to prior radiotherapy status (radiotherapy: n=8; no radiotherapy: n=47; P=0.63). **(D)** OS according to prior chemotherapy status (chemotherapy: n=28; no chemotherapy: n=28; P=0.07). **(E)** OS based on surgical margin status (R0/R1: n = 45; R2: n =11; P =0.87). **(F)** OS stratified by age group (<60 years: n=39; ≥60 years: n=17; P=0.04).

Univariate analysis revealed significant differences in overall survival based on tumor differentiation (WDLPS vs. DDLPS, *P* < 0.05) and FNCLCC grade (grade I: 181.7 months, grade II: 90.3 months, grade III: 84.0 months; *P* < 0.05) (**Figures 2A, B**). In contrast, age, prior radiotherapy, chemotherapy, and surgical margin status were not significantly associated with survival outcomes (**Figures 2C–F**).

Multivariate Cox proportional hazards analysis identified age (hazard ration [HR]=2.92, *P* = 0.04), pathological subtype (HR=11.42, *P* = 0.02), FNCLCC grade (HR=2.39, *P* = 0.04), and frequency of surgical intervention (HR=0.54, *P* = 0.01) as independent prognostic factors (**Table 2**). Sex, history of prior therapies, resection margin status, and postoperative complications did not demonstrate statistically significant associations with overall survival.

**Table 2:** Multivariate Cox proportional hazards analysis of factors associated with OS.

Variable	Hazard ratio	95% CI	*P* value
Gender	1.8	0.68 ~ 4.64	0.24
Age	2.92	1.05 ~ 8.11	0.04
Chemotherapy	2.89	0.92 ~ 9.06	0.07
Radiotherapy	0.067	0.15 ~ 3.19	0.63
Resection margin	1.09	0.40 ~ 2.93	0.87
WDLPS/DDLPS	11.42	2.43 ~ 53.69	0.02
FNCLCC grade	2.39	1.05 ~ 5.40	0.04
Frequency of surgery	0.543	0.34 ~ 0.89	0.01
Morbidity	2.13	0.75 ~ 6.07	0.17

CI, Confidence Intervals.

**Figure 3:**
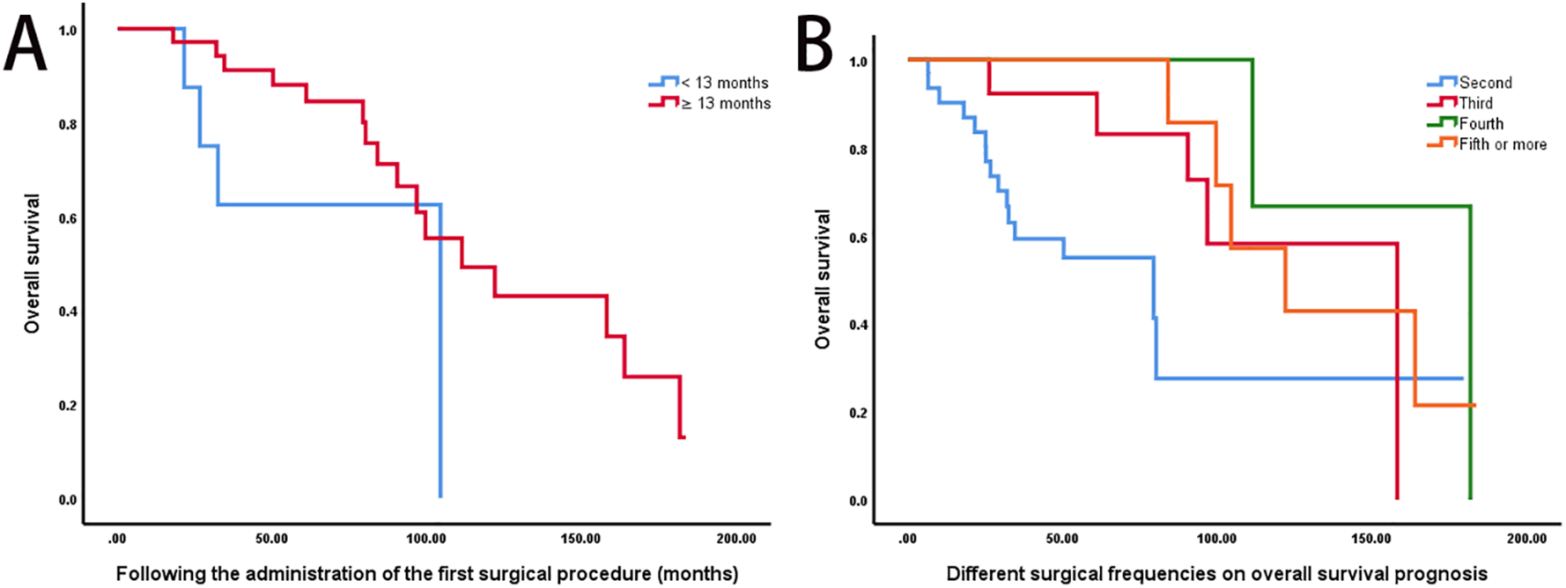
Impact of recurrence timing and surgical frequency on survival outcomes. **(A)** A shorter interval (≤13 months) between the primary and second surgeries was associated with significantly poorer OS compared to delayed recurrence (>13 months) (log-rank P < 0.05). **(B)** Kaplan-Meier OS curves stratified by surgical frequency: 2 surgeries (n = 31, median OS: 79.3 months), 3 surgeries (n = 13, 158.0 months), 4 surgeries (n = 5, 181.7 months), and ≥5 surgeries (n = 7, 121.9 months) (log-rank P < 0.05).

Patients who required a second surgical intervention within 13 months of the initial operation had significantly poorer OS compared to those with later recurrences (*P* < 0.05; **Figure 3A**). Additionally, OS differed varied significantly across groups stratified by the number of surgical resections (*P* < 0.05; **Figure 3B**).

### Morbidity and mortality

SAEs were observed in 10.7% of patients (n=6), including venous catheter-related infections (10.7%, n=6), anastomotic leakage (5.4%, n=3), and postoperative hemorrhage (5.4%, n=3). less frequent complications included pancreatic fistula, incisional infection, or intra-abdominal infection, each occurring in 1.9% of patients (n=1) (**Table 3**). No 30-day postoperative mortality was reported.

**Table 3:** Incidence of postoperative complications.

Variables	n	%	Clavien-Dindo grade
Anastomotic leakage	3	5.4%	III
Hemorrhage	3	5.4%	III
Incisional infection	1	1.9%	II
Venous catheter-related infection	6	10.7%	II
Pancreatic fistula	1	1.9%	III
Intra-abdominal infection	1	1.9%	III

## Discussion

This study represents one of the most comprehensive analyses to date on survival outcomes in patients undergoing multiple surgical resections for locally recurrent RPS. Our findings demonstrate that iterative surgical interventions can significantly improve OS in carefully selected patients, particularly those with WDLPS and lower FNCLCC grades. Notably, median OS increased with repeated surgeries,peaking at four resections (181.7 months), before declining thereafter. This trend may reflect the complex interplay between the potential benefits of surgery and the progressively aggressive biological behavior of recurrent tumors, rather than a strict limit on surgical efficacy.

The progressive shortening of recurrence-free intervals with each subsequent surgery underscores the increasing biological aggressiveness of recurrent RPS, which likely contributes to the diminishing survival benefit despite ongoing surgical efforts.

Bagaria et al. (14) conducted a detailed review on multiply recurrent retroperitoneal liposarcoma (RPLPS), emphasizing the distinct clinical behaviors of histological subtypes. WDLPS was associated primarily with local recurrence and low metastatic potential, whereas DDLPS had higher rates of both local recurrence and distant metastasis, particularly to the lungs. Our findings are consistent with these observations, tumor differentiation as a key determinant of survival.

Wang et al. (15) further compared outcomes across primary RPS, first recurrence (RPS-Rec1), and ≥2 recurrences (≥RPS-Rec2). Patients with RPS-Rec1 who achieved macroscopically complete resection (MCR) had OS and progression-free survival (PFS) comparable to those with primary disease and superior to those with multiple recurrences. Similarly, our data show that OS improves with 2–4 surgeries but declines thereafter, suggesting that early recurrent disease may retain favorable biological characteristics and resectability, iterative surgeries more beneficial.

Data from the Memorial Sloan Kettering Cancer Center (MSKCC) reported a median OS of 28 months after the first recurrence, with resectability decreasing significantly after subsequent relapses (16). In a study by Lochan et al. (17), which included 22 patients with locally recurrent RPS, those who underwent repeat resections had markedly improved outcomes. Median survival after the second surgery was 53.0 months, significantly longer than the 30.0 months observed in patients who did not undergo further surgery (*P <*0.05). These findings support 677 the potential survival benefit of re-resection in selected cases (18). Similarly, the Transatlantic Australasian Retroperitoneal Sarcoma Working Group (TARPSWG) reported a median OS of 49.0 months following second recurrence in patients undergoing surgery (19).

While these previous studies primarily focused on early recurrences, our study extends the evidence base to patients who underwent up to eight surgeries, demonstrating that repeated resections can offer survival benefits even in advanced recurrent disease. However, repeat surgery is technically demanding, particularly due to altered abdominal anatomy and adhesions, posing significant challenges, especially in less experienced centers (17, 19–22).

In our cohort, most patients underwent two surgeries, with a subset undergoing three or more. A clear survival benefit was observed with repeated surgeries, particularly when recurrence occurred after a longer interval. Importantly, the recurrence interval shortened with each subsequent relapse, suggesting that disease control becomes more difficult over time—likely due to more aggressive tumor biology, reduced efficacy of available therapies, and the cumulative impact of surgery on patient condition (11, 23).

Univariate analysis identified tumor differentiation and FNCLCC grade as significant prognostic indicators. WDLPS conferred a longer median OS than DDLPS. Similarly, patients with FNCLCC grade I tumors had better outcomes than those with grades II or III. Prior radiotherapy, chemotherapy, and surgical margin status did not significantly impact OS, consistent with previous reports (2, 9, 21, 22).

The timing of recurrence was also a critical factor. Patients who required a second surgery within 13 months of their initial operation had significantly worse survival, indicating that early recurrence is a strong predictor of poor prognosis. Stratifying patients by surgical frequency revealed significant survival differences, reinforcing the value of surgical resection in selected patients with recurrent RPS (17, 24).

This study has limitations, primarily related to its retrospective design and potential selection bias. Patients who underwent multiple resections likely had better performance status or less aggressive disease. Although multivariate analysis accounted for known prognostic factors (e.g., grade, resection status), unmeasured variables such as patient fitness and surgeon experience may have influenced outcomes. Prospective studies are warranted to further validate these finding and refine patient selection criteria.

Our results support a strategy of aggressive but selective surgical management for recurrent RPS, especially in cases of WDLPS and FNCLCC grade I/II tumors. Nonetheless, the diminishing returns observed beyond four resections and the increasing risks of complications—such as anastomotic leakage and infections —highlight the need for individualized treatment planning. Future research should explore the role of molecular profiling in risk stratification and investigate multimodal strategies to enhance disease control, particularly for patients with unresectable or high-grade disease.

## Conclusion

In conclusion, for patients with locally resectable recurrent retroperitoneal sarcoma, iterative surgical intervention can offer meaningful survival benefits in carefully selected cases. Our findings suggest that repeated resections—particularly up to the fourth surgery—are associated with improved survival outcomes, especially in patients With WDLPS and lower FNCLCC grades. However, patient selection must be individualized, balancing the potential benefits against the risks of surgery and the biology of the disease. Prospective, multicenter trials comparing surgical versus systemic management approaches are essential to establish evidence-based guidelines for the optimal treatment of recurrent RPS.

## Data Availability

The raw data supporting the conclusions of this article will be made available by the authors, without undue reservation.
